# Photothermal Polymer Nanocomposites of Tungsten Bronze Nanorods with Enhanced Tensile Elongation at Low Filler Contents

**DOI:** 10.3390/polym11111740

**Published:** 2019-10-24

**Authors:** Byoungyun Jeon, Taehyung Kim, Dabin Lee, Tae Joo Shin, Kyung Wha Oh, Juhyun Park

**Affiliations:** 1 School of Chemical Engineering and Materials Science, Institute of Energy Converting Soft Materials, Chung-Ang University, Seoul 06974, Korea; jone1993@naver.com (B.J.); acacac97@naver.com (T.K.); imdabin@naver.com (D.L.); 2 UNIST Central Research Facilities & School of Natural Science, Ulsan National Institute of Science and Technology (UNIST), Ulsan 44919, Korea; tjshin@unist.ac.kr; 3 Department of Fashion Design, College of Art, Chung-Ang University, Seoul 06974, Korea

**Keywords:** tungsten bronzes nanorods, nanocomposites, ethylene propylene diene monomer rubber, photothermal, tensile elongation

## Abstract

We present polymer nanocomposites of tungsten bronze nanorods (TBNRs) and ethylene propylene diene monomers (EPDM). The combination of these components allows the simultaneous enhancement in the mechanical and photothermal properties of the composites at low filler contents. The as-synthesized TBNRs had lengths and diameters of 14.0 ± 2.4 nm and 2.5 ± 0.5 nm, respectively, and were capped with oleylamine, which has a chemical structure similar to EPDM, making the TBNRs compatible with the bulk EPDM matrix. The TBNRs absorb a wide range of near-infrared light because of the sub-band transitions induced by alkali metal doping. Thus, the nanocomposites of TBNRs in EPDM showed enhanced photothermal properties owing to the light absorption and subsequent heat emission by the TBNRs. Noticeably, the nanocomposite with only 3 wt% TBNRs presented significantly enhanced tensile strain at break, in comparison with those of pristine EPDM, nanocomposites with 1 and 2 wt % TBNRs, and those with tungsten bronze nanoparticles, because of the alignment of the nanorods during tensile elongation. The photothermal and mechanical properties of these nanocomposites make them promising materials for various applications such as in fibers, foams, clothes with cold weather resistance, patches or mask-like films for efficient transdermal delivery upon heat generation, and photoresponsive surfaces for droplet transport by the thermocapillary effect in microfluidic devices and microengines.

## 1. Introduction

Some materials, when irradiated with long-wavelength near-infrared light (NIR, 780–3000 nm), adsorb and remit the energy as heat. These so-called “photothermal” materials include conducting, semiconducting, and magnetic materials, for example, gold nanomaterials that show surface plasmon resonance [1,2], graphene oxides [3,4,5], donor–acceptor-type conjugated polymers [6,7,8], tungsten bronze [9,10,11], and iron oxide [12,13,14] nanomaterials with band gap transitions. As mentioned, these photothermal materials can absorb NIR light, which has a lower energy and longer wavelength than ultraviolet or visible light and is harmless to the human body; furthermore, NIR light is not scattered significantly by tissue, allowing deep tissue penetration. Therefore, these materials have been extensively investigated for a variety of applications such as in photoacoustic imaging, photothermal therapy for cancer ablation, and heating.

Recently, photothermal nanomaterials and films have been highlighted as a frontier thermal-based technology for transdermal delivery systems [15,16,17,18,19] and photoresponsive surfaces for the manipulation of microscale liquid droplets [20,21,22,23] in microfluidics and microengines, as well as a conventional technology for fibers, films, and clothes with cold weather resistance, steam generation and military applications [24,25,26]. For example, soft, stretchable masks and patches containing drugs can be placed in contact with the skin, providing fluidity to the lipid layers in the stratum corneum (the outermost layer of the epidermis, which is the main route for drug/cosmetic delivery), when heat is provided and the skin temperature increases [19]. In addition, the mass diffusivity of drug/cosmetic components increases with skin temperature, thereby enhancing mass transport across the stratum corneum [19]. In addition, local NIR irradiation of the surface of composites containing NIR-absorbing photothermal materials induces a dynamic temperature gradient, thus transporting liquid droplets by asymmetrical droplet deformation and Marangoni flow inside the droplets, which is significant for mass transport in microfluidic devices and microengines [20,21,22,23]. Furthermore, the warming of outdoor clothes, shoes, and wetsuits by photothermal materials irradiated with sunlight would be beneficial in harsh and cold environments [24,25,26]. Although there are many obvious advantages of deformable photothermal polymer nanocomposites for new technologies such as patches, masks, or bandages, there are only handful examples of polymer nanocomposites that contain efficient photothermal materials. Furthermore, currently, only joule heating by electrical conductors [27], laser irradiation [28], or the distribution of photothermal materials directly on the skin [29] or in hydrogels [30] are available. This lack of polymer nanocomposites is primarily because nanosized photothermal materials with hydrophobic surfaces are required for compatible mixing with polymers, for example, photothermal Fe_3_O_4_ nanoparticles capped with polymer chains and embedded in polydimethylsiloxane [20]. Thus, the development of stretchable, deformable polymer nanocomposites containing efficient photothermal materials is necessary for future photothermal technologies.

In this study, we pay special attention to the effectiveness of photothermal tungsten bronze nanomaterials (TBNs) that include tungsten bronze nanorods (TBNRs) and tungsten bronze nanoparticle (TBNPs), and their polymer nanocomposites. Tungsten bronzes (TB, M*_x_*WO_3_) are tungsten-trioxide-based materials doped with alkali metals (M = Li, Na, K, or Cs) [31,32,33,34]. The alkali metals share their valence electrons with the conduction band of tungsten trioxide when intercalated into the framework formed of corner-sharing WO_6_ octahedrals, thereby forming surface plasmon polaritons of free electrons and sub-bands in the conduction band of tungsten trioxide [31,32,33,34]. Thus, TBNs exhibit high absorption in the NIR wavelength range owing to sub-band transitions and have adjustable electrical properties, being semiconductors at low concentrations of metal ions and conductors at high concentrations [35]. Thus, because of their semiconducting properties and indirect bandgap at low concentrations of metal ions, these materials release heat upon the relaxation of the absorbed energy via radiationless decay rather than photoluminescence [36]. This photothermal property of tungsten bronzes is advantageous for biomedical applications, for example, cancer cells can be killed by administering the TBNs to the tumor and irradiating the site with NIR light; the NIR energy is converted into heat, thus destroying the malignant cells in situ [9,10,37]. However, this application has not yet been demonstrated for polymer nanocomposites.

**Figure 1:**
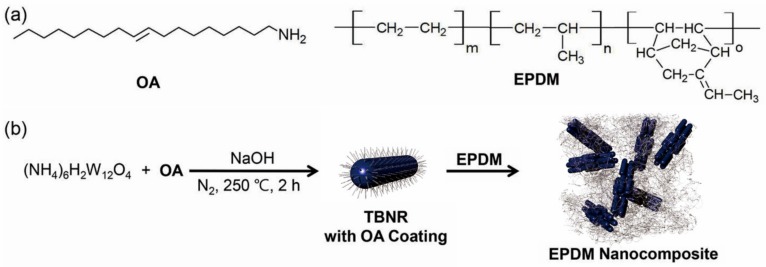
(**a**) Chemical structures of oleylamine (OA) and ethylene propylene diene monomers (EPDM). (**b**) Schematic illustration of preparation of tungsten bronze nanorods (TBNRs) surrounded by OA and their nanocomposite with EPDM.

Herein, we report nanocomposites of an elastomer material with TBNRs that were synthesized via the thermal decomposition of a precursor in oleylamine (OA) and the simultaneous enhancement of their mechanical and photothermal properties. The objectives of this study are to demonstrate a model polymer nanocomposite bearing photothermal tungsten bronze nanorods and to investigate the influence of nanorod distribution on the photothermal and mechanical properties of the nanocomposite. We have characterized the structure, composition, morphology, and optical properties of the TBNRs. We have also examined the successful distribution of TBNRs capped with OA in an ethylene-propylene-diene monomer (EPDM) matrix (Figure 1), a terpolymer of ethylene, propylene, and a minor amount of a non-conjugated diene monomer (Figure 1a). Unlike current approaches for EPDM-based nanocomposites that mainly focus on the enhancement of their mechanical properties [38,39,40,41], we demonstrate the straightforward synthesis of TBNRs with efficient NIR absorption properties via a simple one-pot process, and the enhancement in both the photothermal and mechanical properties of the EPDM nanocomposites (Figure 1b) is promising for future nanobiotechnological applications. Different from current solvothermal technologies for TBNR synthesis that produce nanorods with micrometer-scale lengths and no surface alkyl coating [42,43], our one-pot process results in TBNRs with a length of a few tens of nanometers and a surface alkyl coating. The photothermal properties, tensile strength, elongation, and storage modulus of the resulting nanocomposites are measured and compared with those of nanocomposites based on TBNPs because rod-like or wire-like fillers are beneficial for enhancing the mechanical properties, as shown in polymer composites with carbon nanofibers [44,45] and zinc oxide nanorods [46].

## 2. Materials and Methods

### 2.1. Materials

Ammonium metatungstate hydrate (AMT, (NH_4_)_6_H_2_W_12_O_40_)·xH_2_O, MW = 2956.3 g/mol) and OA (>70%) were purchased from Sigma–Aldrich (St. Louis, MO, USA). Toluene and acetone were purchased from DaeJung Chemical (Siheung-si, Gyeonggi-do, South Korea). Sodium hydroxide (NaOH) was purchased from Samchun Chemical (Seoul, South Korea). EPDM was produced from a bio-based feedstock, of which the ethylene (derived from ethanol from sugarcane) was provided by LANXESS (Keltan^®^ Eco 6950C, ethylene 44 wt %, propylene 47 wt %, ethylene norbornene 9 wt%, Mooney viscosity = 65 MU at 125 °C, Cologne, Germany). All the reagents used in this study were used as received.

### 2.2. TBN Synthesis

The TBNRs were synthesized according to our previously reported TBN synthesis via the thermal decomposition of AMT in OA [47]. In brief, 0.1 mmol (0.2956 g) of AMT and 16 mL of oleylamine were added to a three-necked round flask and stirred for 1 h to prepare a slurry. Then, 0.396 mmol (0.01584 g, 33 mol% with respect to W) of NaOH was added, and the mixture was stirred for an additional 1 h. A reflux condenser was connected to the flask, and nitrogen gas was injected for 1 h to refresh the atmosphere in the flask with the inert gas. Thereafter, the mixture was gradually heated to 250 °C and stirred for 2 h under nitrogen. After 2 h of reaction, the reaction mixture was naturally cooled to room temperature. The precipitate was collected by centrifuging at 8000 rpm for 15 min with excess acetone to remove the excess OA. The collected precipitate was re-dispersed in acetone and centrifuged twice at room temperature to obtain TBNR powder. This powder was re-dispersed in toluene for characterization. Cesium TBNP was also synthesized following this procedure but using CsOH (33 mol% with respect to W) rather than NaOH as the alkali metal source.

### 2.3. Preparation of TBNR/EPDM Nanocomposite Films

Three grams of EPDM was dissolved in toluene at 15 wt % and 110 °C. A TBNR dispersion in toluene was added to the EPDM solution with stirring at 150 rpm. Solutions were prepared at four TBNR concentrations (0, 1, 2, and 3 wt %), poured into Petri dishes, and dried overnight under a vacuum at room temperature. We used a solution mixing process followed by solvent removal, instead of a melt extrusion process to save the used amounts of TBNRs in our model study and to ensure a complete mixing of EPDM and TBNR at a processing temperature lower than that of the extrusion process.

### 2.4. Characterization

The morphology and structure of the TBNs were characterized using high-resolution transmission electron microscopy (HR-TEM, JEM3010, JEOL, Akishima, Japan). The sizes of the TBNs were estimated by analyzing the TEM images using a Gatan Microscopy Suite (Gatan Inc., Pleasanton, CA, USA). The crystal structures of the TBNs were verified by X-ray diffractometry (XRD, Bruker-AXS NEW D8 Advance, Billerica, MA, USA). The chemical states of the elements in the TBNs were determined using X-ray photoelectron spectroscopy (XPS) with monochromated Al Kα X-rays (1486.6 eV, K-Alpha+ XPS system, ThermoFisher Scientific, Waltham, MA, USA). The binding energies were calibrated with respect to the C 1s core level position at 284.8 eV as an internal reference. The survey and narrow scan spectra were obtained with analyzer pass energies of 200 and 40 eV, respectively. The compositions of the TBNs were further verified by energy dispersive spectroscopy (EDS, NORAN System 7, ThermoFisher Scientific). The presence of OA on the surface of the nanorods was verified using Fourier transform infrared spectroscopy (FT-IR, Nicolet 6700, ThermoFisher Scientific) and thermogravimetric analysis (TGA, TGA-2050, TA Instruments, New Castle, DE, USA). TGA analyses were carried out under a nitrogen atmosphere at a heating rate of 10 °C/min. Ultraviolet-visible (UV-Vis) absorption spectra were obtained with a UV-Vis spectrometer (V-670, JASCO, Tokyo, Japan) in the range of 300–2100 nm. Structural analysis under tensile elongation using two-dimensional (2D) wide-angle and small-angle X-ray scattering (2D WAXS and SAXS, respectively) was conducted at a synchrotron facility (6D UNIST-PAL beamline of PLS-II at Pohang Accelerator Laboratory, Republic of Korea).

### 2.5. Photothermal and Mechanical Analysis

The photothermal properties of the EPDM–TBN nanocomposite films were determined using a solar simulator with white light (100 W, PEC-L01, Peccell Technologies Inc., Yokohama, Japan), which was applied to the films at a distance of 20 cm for 1 h, and NIR images were captured every 20 s for the first 10 min, and, then, every 10 min for the remaining time using a NIR camera (TG165, FLIR Systems, Wilsonville, OR, USA). Then, the white light was turned off, and NIR images of the nanocomposite films were recorded for another 1 h every 10 min to examine the thermal storage properties. To monitor the photothermal increase of the rubber nanocomposite containing 3 wt % TBNR, the film was attached to the skin of the forearm of a human subject and NIR light from a commercial medical NIR lamp (Sunglim Medical Devices Co., Seoul, South Korea, Model YL-250 with a 250 W NIR bulb from Phillips Co., Amsterdam, Netherlands was used to illuminate the sample. The phase transition behaviors of the TB nanocomposite films were measured using differential scanning calorimetry (DSC, DSC214 Polyma, NETZSCH, Selb, Germany) under a nitrogen gas atmosphere. Each sample (approximately 10 mg) was placed in an aluminum pan and measured in the temperature range of −125 to 160 °C at a heating rate of 20 °C/min, under a nitrogen atmosphere. To remove the effects of the thermal history, data recorded in the second run were used for the analysis. Dynamic mechanical analysis (DMA, Q800, TA Instruments, New Castle, DE, USA) were carried out in tension mode at a frequency of 1 Hz in the temperature range −100 to 100 °C at a heating rate of 3 °C/min. Tensile tests were carried out by using a universal testing machine (UTM, WL2100, WITHLAB, Gunpo-si, Gyeonggi-do, South Korea) at room temperature and an extension rate of 500 mm/min with a load cell of 20 N. The dimensions (width × thickness) of the nanocomposite thin films with 0, 1, 2, and 3-wt% TBNRs were (10.22 ± 0.50) × (0.29 ± 0.05), (10.01 ± 0.40) × (0.23 ± 0.03), (10.18 ± 1.00) × (0.29 ± 0.08), and (9.75 ± 1.50) × (0.25 ± 0.05) mm^2^, respectively. Five specimens of each pristine EPDM and nanocomposite film were measured.

## 3. Results

### 3.1. Structures, Morphologies, and Compositions of TBNs

**Figure 2:**
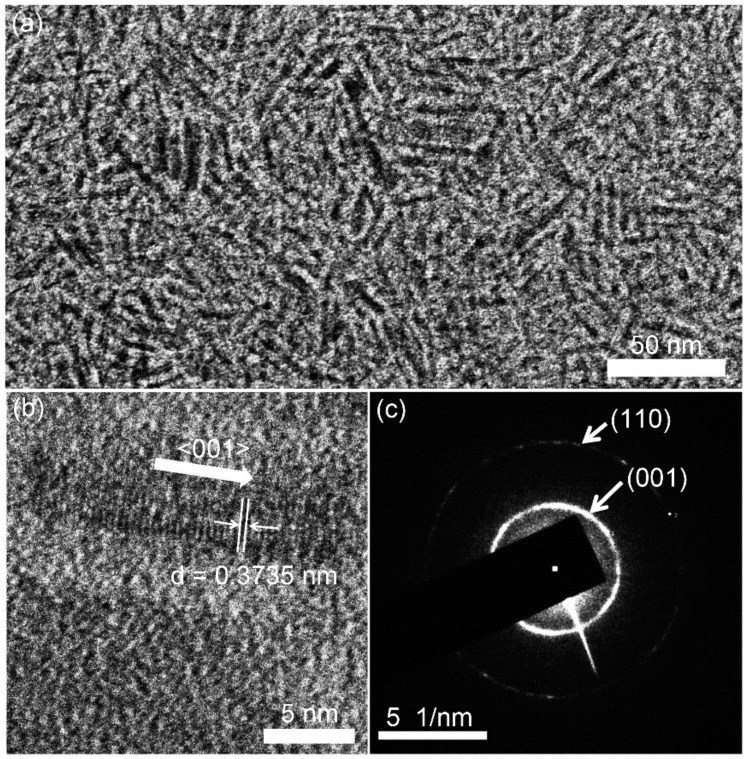
(**a**) Transmission electron microscopy (TEM) and (**b**) high-resolution transmission electron microscopy (HR-TEM) images, and (**c**) selected area electron diffraction (SAED) pattern of Na_0.33_WO_3_ TBNRs.

The Na_0.33_WO_3_ TBNRs and Cs_0.33_WO_3_ TBNPs were synthesized via the thermal decomposition of AMT with alkali metals and OA following our previously reported procedure [47]. Unlike our previous report that focused on the enhancement of NIR absorption properties by synthesizing TBNPs of a quaternary compound (Na_0.11_Cs_0.22_WO_3_), in this study, we elucidated the morphologies of tungsten bronze ternary compounds obtained by thermal decomposition and investigated the effect of the morphology on the nanocomposite properties. Although the crystal structures of Na_x_WO_z_ and Cs_y_WO_z_ (ternary tungsten bronzes prepared via the thermal decomposition route with OA and AMT) have already been reported [47], their morphologies and properties were not examined in detail. The structure and morphology of the TBNs were verified by using XRD, HR-TEM, and selected area electron diffraction (SAED). The XRD patterns of Na_0.33_WO_3_ and Cs_0.33_WO_3_ match those of cubic Na_0.11_WO_3_ (Powder Diffraction File (PDF) 01-075-0057, space group = Pm-3m, a = 3.735 Å) and cubic (Cs_2_O)_0.44_W_2_O_6_ (PDF 00-047-0566, space group = Fd-3m, a = 10.274 Å), respectively (Figure S1a,b) and are almost identical to the XRD patterns of the ternary compounds of Na_x_WO_z_ and Cs_y_WO_z_ described in our previous report [47]. The Na_0.33_WO_3_ had a nanorod morphology of length 14.0 ± 2.4 nm and diameter of 2.5 ± 0.5 nm (aspect ratio ≈ 6), as estimated by image analysis (Figure 2a). The nanorod growth occurred along the <001> direction, as shown by the high intensity of the diffraction peak of the (001) plane (Figure 2b and Figure S1a). The clear diffraction rings of the (001) and (110) planes are also apparent in the SAED patterns (Figure 2c). In comparison, Cs_0.33_WO_3_, which has the pyrochlore structure in the space group Fd-3m, presents a particulate crystal morphology, having an average particle size of 7.8 ± 1.9 nm, as shown in Figure S1c. The difference in the crystal structures likely originates from the size difference between Na^+^ and Cs^+^ ions. The metal tungstate anion ([α-H_2_W_12_O_40_]^6−^) produced by the thermal decomposition of AMT exhibits a cubic pyrochlore structure having defects at the 8b, 16d, and 32e positions [48,49]. It is likely that the large Cs^+^ ions are intercalated at the 8b and 32e sites, stabilizing the structure with stacking layers along the [311] direction, whereas the small Na^+^ ions are inserted in the 16d positions with stacking layers along the [001] direction [48].

**Figure 3:**
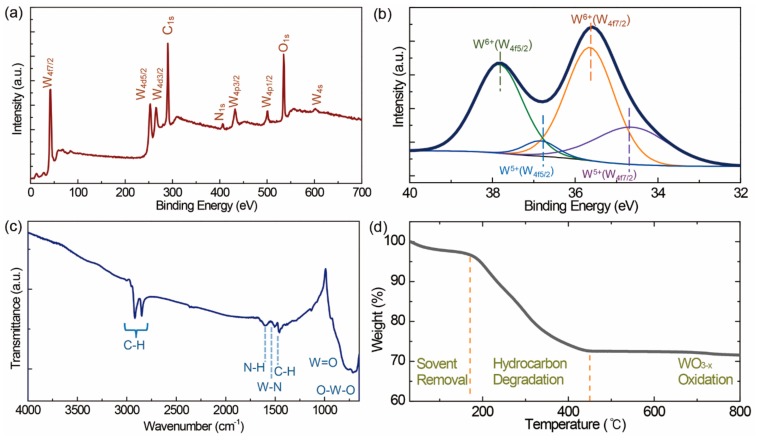
Na_0.33_WO_3_ TBNRs with OA coating. Compositional analysis: (**a**) full scan X-ray photoelectron spectroscopy (XPS) spectra and (**b**) W_4f_ core-level XPS spectra. (**c**) Fourier transform infrared spectroscopy (FT-IR) spectrum and (**d**) thermogravimetric analysis (TGA) thermogram.

The chemical composition and valence states of the Na_0.33_WO_3_ TBNRs were identified by XPS (Figure 3a,b) and EDS (Figure S2 and Table S1) measurements. The composition of the TBNRs can be estimated by deconvoluting the W_4f_ peak into two spin-orbit doublets: W_4f7/2_ and W_4f5/2_, which have a separation of 2.2 eV. The main fitted peaks at 37.88 and 35.68 eV are attributed to the W^6+^ oxidation state. The second doublet (with lower binding energies 36.88 and 34.68 eV) is due to the release of electrons from the W_4f5/2_ and W_4f7/2_ core levels of the W^5+^ oxidation state. As the peaks from the W^5+^ state originate from the reduction of W^6+^ by electron transfer from the doped sodium, the mole fraction of doped-sodium can be estimated by integrating the doublets of both the W^6+^ and W^5+^ states (W^6+^O_3_ → M_x_W_1-x_^6+^W_x_^5+^O_3_). The intercalated mole fraction of alkali metal in the Na_0.33_WO_3_ TBNRs estimated from the XPS spectra is 0.33, which coincides with the mole fraction of the sodium source in the reaction mixture. For Cs_0.33_WO_3_ TBNPs, the mole fraction of cesium was also estimated to be 0.33 relative to tungsten.

### 3.2. Na_0.33_WO_3_ TBNRs with OA Coating

One of the key factors to the compatibility of Na_0.33_WO_3_ TBNRs in an EPDM matrix is the surface OA that has an identical chemical structure with EPDM. The presence of OA on the surfaces of the TBNRs was verified by FT-IR (Figure 3c) and TGA (Figure 3d) measurements. The characteristic IR bands for vinyl C–H stretching (2999 cm^−1^), aliphatic asymmetric (2918 cm^−1^), and symmetric (2849 cm^−1^) C–H stretching, N–H bending (1601 cm^−1^), W–N vibration (1504 cm^−1^), and CH3 bending (1456 cm^−1^) peaks [47,50] establish the presence of OA in the Na_0.33_WO_3_ TBNRs. Moreover, the IR spectrum in the low wavenumber range (below 1000 cm^−1^) exhibits characteristic W=O stretching (932 cm^−1^) and O–W–O stretching (760 and 714 cm^−1^) bands [51,52]. Therefore, it is apparent that OA was attached to the Na_0.33_WO_3_ TBNR surfaces. The TGA thermogram (Figure 3d) shows two mass losses in the temperature ranges 150−450 °C and 630–740 °C. The first mass reduction of 3.3% below 170 °C is assigned to solvent evaporation. The second mass reduction of 24.0% between 170 and 450 °C originates from the decomposition of OA hydrocarbon chains, and the subsequent third mass reduction of 0.8% between 630 and 740 °C is related to the oxidation of WO_3-x_. The residual weight percentage was 71.6 wt%. Owing to the surface hydrocarbons, the TBNRs were readily dispersed in a non-polar organic solvent, toluene.

### 3.3. EPDM Nanocomposite Films with TBNs and Their Photothermal Properties

**Figure 4:**
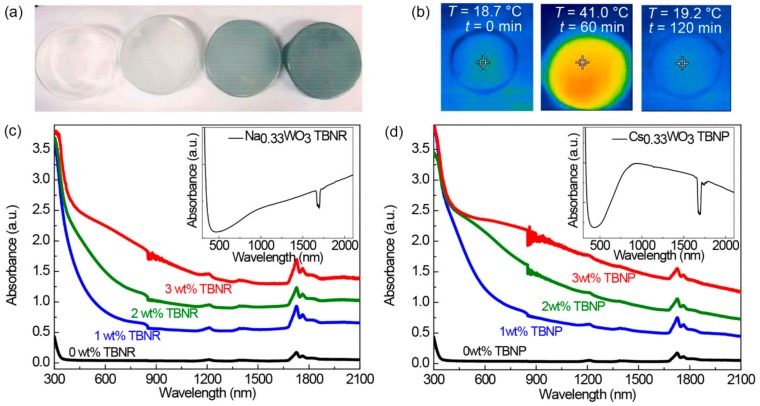
(**a**) Optical images of EPDM nanocomposite films with 0, 1, 2, and 3 wt % Na_0.33_WO_3_ TBNRs and (**b**) representative near-infrared light (NIR) images of a nanocomposite film with 3 wt % Na_0.33_WO_3_ TBNRs under white light irradiation at 0 and 60 min and after the light was turned off for 1 h (*t* = 120 min). UV absorption spectra of nanocomposite films containing (**c**) Na_0.33_WO_3_ TBNRs and (**d**) Cs_0.33_WO_3_ tungsten bronze nanoparticle (TBNPs). Those of the Na_0.33_WO_3_ TBNR and Cs_0.33_WO_3_ TBNP dispersions in toluene are shown in the insets of (**c**) and (**d**), respectively (the break at approximately 850 nm is due to a mirror change in the spectrometer).

The nanocomposites of EPDM and Na_0.33_WO_3_ TBNRs were readily formed by mixing a toluene solution of EPDM and a toluene dispersion of Na_0.33_WO_3_ TBNRs, followed by solvent removal after pouring the mixed solution into petri dishes. As the concentration of the Na_0.33_WO_3_ TBNRs in the EPDM matrix increased to 3 wt%, the color of the transparent nanocomposite, which was colorless for the 0 wt % Na_0.33_WO_3_ TBNR film, became dark blue (Figure 4a). The temperatures of the nanocomposite films were measured by an NIR camera under white light irradiation produced using a solar simulator for 1 h, followed by another measurement for 1 h after turning off the light. The representative NIR images of the EPDM/Na_0.33_WO_3_ TBNR (3 wt %) nanocomposite film captured at *t* = 0, 60, and 120 min are shown in Figure 4b. The temperature of the EPDM nanocomposite film with 3 wt% TBNRs increased to 41 °C, followed by a temperature decrease when the light was turned off. The observed photothermal temperature increase of the EPDM nanocomposite should originate from the efficient light absorption of the Na_0.33_WO_3_ TBNRs and Cs_0.33_WO_3_ TBNPs in EPDM. The absorption intensities of the nanocomposite films incorporating Na_0.33_WO_3_ TBNRs and Cs_0.33_WO_3_ TBNPs (shown in Figure 4c,d, respectively) increase correspondingly with the increasing concentration of TBNs over the measurement range from 300 to 2100 nm. The absorption spectra of the Na_0.33_WO_3_ TBNR and Cs_0.33_WO_3_ TBNP dispersions in toluene (inset images of Figure 4c,d) exhibit strong UV absorption below 400 nm and NIR absorption above 780 nm owing to the inter-band transition from the valence band of oxygen (O 2p) to the conduction band of tungsten (W 5d) and sub-band transition of TBNs, respectively. The NIR absorption originates from the pairing of the surface plasmon polaritons and the electric field of the NIR light [31,32,33]. It should be mentioned that the absorption band at approximately 1700 nm originates from the residual toluene in the films. We did not completely remove the residual solvent because high-vacuum treatment of the samples at elevated temperatures to remove the solvent completely causes the formation of bubbles in the films, resulting in non-neat films that are not suitable for mechanical testing, although they may be suitable for foaming applications. Rather, we applied an identical solvent removal process to all the films (drying in a vacuum at room temperature) to obtain neat nanocomposite films, as shown in Figure 4a. A weight loss of 3.3 wt% at 170 °C, the first transition point in the TGA thermogram in Figure 3d, is assigned to the residual solvent.

**Figure 5:**
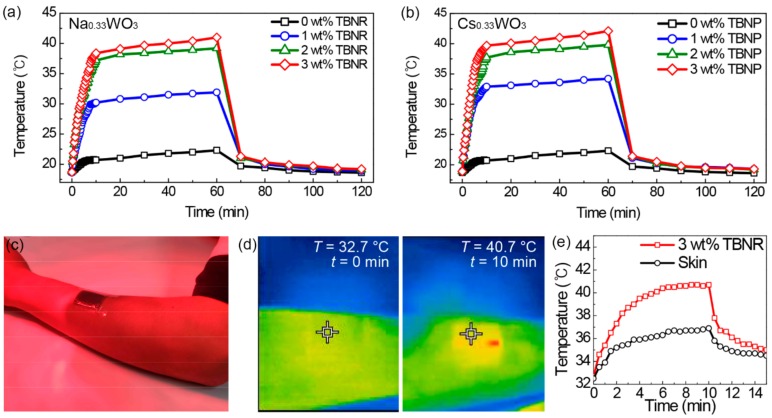
Photothermal temperature increases of nanocomposite films containing (**a**) Na_0.33_WO_3_ TBNRs and (**b**) Cs_0.33_WO_3_ TBNPs under white light irradiation for 60 min, followed by temperature decreases for 60 min after turning off the light. (**c**) An optical image of an arm with a patch of a nanocomposite film with 3 wt % TBNRs under NIR irradiation by a medical NIR lamp, and (**d**) NIR images of the arm at 0 and 10 min under NIR irradiation. (**e**) Photothermal temperature increases of the skin and patch including 3 wt % Na_0.33_WO_3_ TBNRs under NIR light irradiation for 10 min, followed by temperature decreases after turning off the light.

Figure 5a,b show the photothermal temperature increases measured every 20 s for the first 10 min and then every 10 min for the remaining time. There is a drastic increase in the film temperatures over 10 min of white light irradiation; then, the temperature increase slows until 1 h. The temperature increase rates in the first 3 min of the light irradiation were 2.0, 3.3, and 3.5 °C/min for the nanocomposite films with 1, 2, and 3 wt % Na_0.33_WO_3_ TBNRs, respectively (Figure 5a and Table 1). In comparison, that of pristine EPDM was 0.4 °C/min. The maximum temperatures after the 1h light irradiation were proportional to the concentrations of TBNRs, and were 31.9, 39.2, and 41.0 °C for the nanocomposite films with 1, 2, and 3 wt % Na_0.33_WO_3_ TBNRs, respectively; meanwhile, the pristine EPDM exhibited a minimal increase (from 18.7 to 22.3 °C) over 1 h. The nanocomposite films with Cs_0.33_WO_3_ TBNPs presented a photothermal behavior similar to those with Na_0.33_WO_3_ TBNRs (Figure 5b and Table 1). The temperature increase rates and maximum temperatures of the nanocomposite films with 1, 2, and 3 wt % Cs_0.33_WO_3_ TBNPs were 2.9 °C/min and 34.2 °C, 3.3 °C/min and 39.8 °C, and 3.7 °C/min and 42.1 °C, respectively. The nanocomposite films with Cs_0.33_WO_3_ TBNPs exhibited marginally higher increases in the photothermal temperatures than those with Na_0.33_WO_3_ TBNRs, probably owing to the larger surface area of spheres than that of the rods for the heat transfer from the TBN fillers to the EPDM matrix or the difference in the amount of light absorption. Overall, these results show the remarkable photothermal properties of Na_0.33_WO_3_ TBNR and Cs_0.33_WO_3_ TBNP fillers in the EPDM matrix. The temperatures of the nanocomposite films substantially decreased after turning off the light for 10 min, almost attaining room temperature after 1 h, indicating that the thermal storage effect of the TBN fillers was not significant. We further examined the applicability of the rubber nanocomposite film as a patch for transdermal drug/cosmetic delivery by cutting the EPDM film containing 3 wt % Na_0.33_WO_3_ TBNR to a 30 × 30 × 1.5 mm^3^ patch and attaching it to the skin of a human arm, followed by NIR irradiation using a commercially available portable medical NIR lamp (Figure 5c). At 50 cm separation of the NIR bulb at 125 W to the skin, the patch temperature increased from 32.7 to 38.0 °C in 2.5 min, reaching a plateau at 40.7 °C (Figure 5d,e). In contrast, the temperature of the bare skin increased from 32.5 to 36.9 °C after 10 min of NIR irradiation. These results show that the photothermal film temperature can be controlled in a range optimal for transdermal delivery applications in a few minutes.

### 3.4. Phase Transition and Mechanical Behaviors of Nanocomposite Films

**Figure 6:**
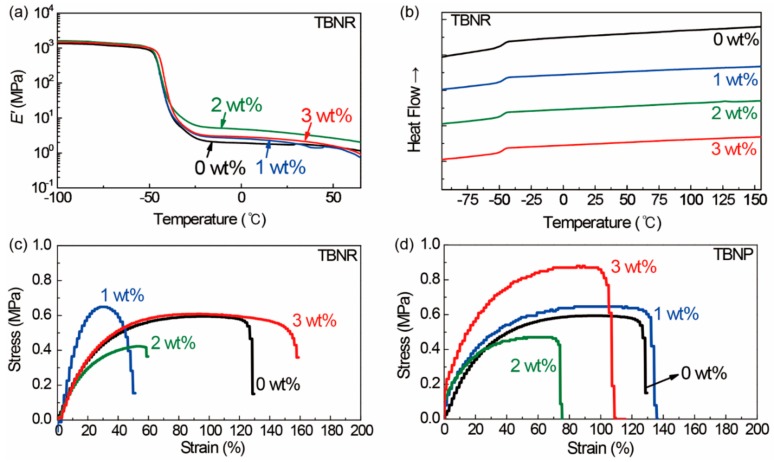
(**a**) Dynamic mechanical analysis (DMA) storage modulus curves and (**b**) differential scanning calorimetry (DSC) thermograms of Na_0.33_WO_3_ TBNR/EPDM rubber nanocomposite films. Tensile stress–strain curves of EPDM nanocomposite films including (**c**) Na_0.33_WO_3_ TBNRs and (**d**) Cs_0.33_WO_3_ TBNPs.

The phase transition behaviors of the nanocomposite films with TBNs were not significantly different from that of the pristine EPDM, and the films exhibited marginally enhanced storage moduli and tensile strengths compared to those of the pristine EPDM, although irregular values were measured for nanocomposites containing 1 and 2 wt % Na_0.33_WO_3_ TBNRs, as discussed in the following paragraphs. The glass transition temperatures (*T*_g_) of the pristine EPDM, estimated from the maximum of tanδ in the DMA measurements, and the second-order transition in the DSC thermogram were −40.4 and −48.3 °C, respectively (Table 1, Figure 6a,b), indicating its amorphous structure without crystalline regions. As estimated by DMA and DSC, the *T*_g_ values of the nanocomposite films with Na_0.33_WO_3_ TBNRs slightly increased to −38.8 and −47.3 °C, respectively, with 3 wt % Na_0.33_WO_3_ TBNRs. Moreover, that of the nanocomposite films with Cs_0.33_WO_3_ TBNPs increased to −46.5 °C at 3 wt % Cs_0.33_WO_3_ TBNPs, as estimated by DSC. These results indicate that the interfacial interaction between OA chains on the TB particle surfaces and EPDM matrix contributes to the enhancement of the rigidity of the nanocomposite films.

The significantly different mechanical behaviors of the nanocomposite films with and without Na_0.33_WO_3_ TBNRs is clear in the tensile stress–strain curves (Figure 6c,d). The averaged tensile strengths and strains are summarized in Table 1, and the representative tensile stress–strain curves that exhibit data most close to the averaged values are plotted in Figure 5c,d. The pristine EPDM film (0 wt % TBNs; Figure 6c,d) exhibit tensile strength and strain of 0.59 ± 0.03 MPa and 120 ± 16%, respectively. When 1 and 2 wt % Na_0.33_WO_3_ TBNRs were composited with EPDM, the resulting nanocomposite films exhibited tensile strengths and strains of 0.55 ± 0.11 MPa and 51 ± 6%, and 0.40 ± 0.06 MPa and 61 ± 18%, respectively; that is, the mechanical properties were severely deteriorated. However, as the concentration of Na_0.33_WO_3_ TBNRs was further increased to 3 wt %, the tensile strength and strain of the resulting nanocomposite film improved significantly to 0.61 ± 0.05 MPa and 165 ± 20%, respectively. On the other hand, the nanocomposite films with Cs_0.33_WO_3_ TBNPs at concentrations of 1, 2, and 3 wt % Cs_0.33_WO_3_ TBNPs exhibited larger tensile strengths than that of the pristine EPDM film. Meanwhile, the tensile strain of the nanocomposite films with 1 wt % Cs_0.33_WO_3_ TBNPs was higher than those of the pristine EPDM, whereas those with 2 and 3 wt % Cs_0.33_WO_3_ TBNPs were lower. The tensile strains were 130 ± 21%, 93 ± 14%, and 98 ± 20% for the nanocomposite films with 1, 2, and 3 wt % TBNPs, respectively.

The low tensile strengths and elongations at 1 and 2 wt % Na_0.33_WO_3_ TBNRs indicate that the Na_0.33_WO_3_ TBNRs in the EPDM matrix formed nanocomposite films with a heterogeneous morphology and were likely to have functioned as sites for fracture development. The storage modulus values of the nanocomposite films at 1 and 2 wt % Na_0.33_WO_3_ TBNRs were 2.14 and 3.97 MPa, respectively, and higher than that of the pristine EPDM film (1.76 MPa). The inverse relation between the tensile strengths and the higher storage modulus is typical of nanocomposite polymers with inorganic fillers, arising because the inorganic fillers in the matrix are regions of high stress, resulting in cohesive failure and reduced strain at break [53]. In comparison, the nanocomposite film with 3 wt % Na_0.33_WO_3_ TBNRs presents a significantly enhanced tensile elongation with a tensile strength comparable to that of the pristine EPDM. The origin of the enhanced tensile strain is elucidated in the later part of this section. It seems that regions between nanorods are likely to function as stress concentration points and the stress might be relieved during the elongational alignment. In contrast to the mechanical behaviors of the nanocomposite films with Na_0.33_WO_3_ TBNRs, those of Cs_0.33_WO_3_ TBNPs show higher tensile strengths at 1, 2 and 3 wt % Cs_0.33_WO_3_ TBNP concentrations than that of the pristine EPDM. The increase in the tensile strengths of the nanocomposite films with increasing Cs_0.33_WO_3_ TBNP concentrations could result from the interfacial interaction between OA chains on the Cs_0.33_WO_3_ TBNP surfaces and the EPDM matrix. Due to the miscibility between OA on the Cs_0.33_WO_3_ TBNP surfaces and the EPDM matrix and the corresponding enhancement in the intermolecular interactions between alkyl chains, it is reasonable to predict that the tensile strength increases with Cs_0.33_WO_3_ TBNP concentration, as also revealed by molecular dynamics simulations of polymer composites with nanoparticles [54].

**Figure 7:**
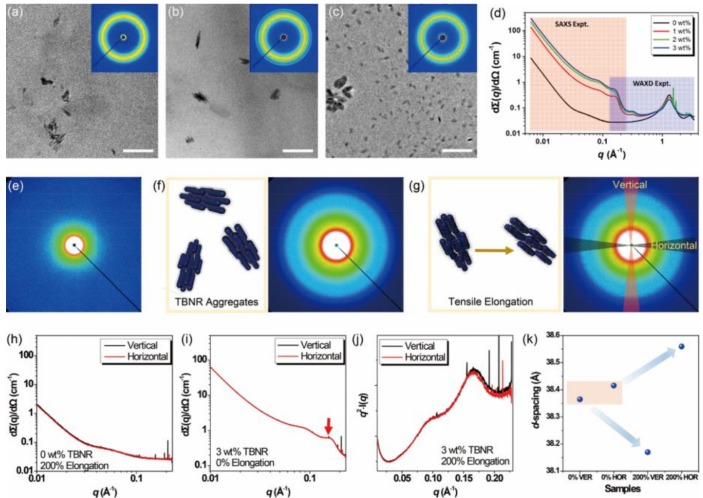
TEM images of EPDM nanocomposites with (**a**) 1, (**b**) 2, and (**c**) 3 wt % Na_0.33_WO_3_ TBNRs. The scale bar is 200 nm. Inlet images are 2D wide-angle X-ray scattering (WAXS) profiles of the nanocomposites in *q* ranges from 0.00 to 2.00 Å^−1^. (**d**) Merged profiles of 1D small-angle X-ray scattering (SAXS) and WAXS of the nanocomposites obtained after circular integration of the 2D images. 2D SAXS profiles of nanocomposites with (**e**) 0 and (**f**) 3 wt % Na_0.33_WO_3_ TBNR at 0% tensile elongation, and (**g**) at 200% tensile elongation in *q* ranges from 0.00 to 0.22 Å^−1^ with schematic illustrations of TBNR aggregates. 1D SAXS profiles obtained by integrating azimuthal cuts of 2D images (vertical: 80–100° and 260–280°, horizontal: 170–190° and −10 to 10°): EPDM nanocomposites with (**h**) 0 wt % Na_0.33_WO_3_ TBNR at 200% elongation and (**i**) 3 wt % at 0% elongation. Enlarged profiles of the peak with a red arrow in (**i**): nanocomposites with 3 wt % TBNRs at (**j**) 200% tensile elongation and (**k**) corresponding *d*-spacing variations of samples.

The origin for the stress–strain behaviors of EPDM nanocomposites with Na_0.33_WO_3_ TBNRs were experimentally investigated by obtaining their cross-sectional TEM images and X-ray scattering profiles with and without uniaxial elongation (Figure 7). The cross-sectional TEM image of the nanocomposite film at 1 wt% Na_0.33_WO_3_ TBNR concentration (Figure 7a) shows an inhomogeneous distribution of Na_0.33_WO_3_ TBNRs in the EPDM matrix, and that at 2 wt % Na_0.33_WO_3_ TBNR shows aggregates of Na_0.33_WO_3_ TBNRs in the EPDM matrix (Figure 7b). In contrast, ellipsoidal particulates were evenly distributed in the EPDM matrix with 3 wt % TBNRs, although with some partial aggregates with a size of a hundred nanometers, as shown in Figure 7c and Figure S3. The size of ellipsoidal particulates is about 46 nm in length and 20 nm in width, indicating that they are also aggregates of Na_0.33_WO_3_ TBNRs. 2D and 1D WAXS profiles of these films are shown in the inset images of Figure 7a–d, showing crystal peaks for the EPDM nanocomposites with 2 and 3 wt % Na_0.33_WO_3_ TBNRs together with an amorphous hollow peak centered at q = 1.30 Å^−1^ (d = 4.83 Å) characteristic of EPDM [55]. A crystalline peak at q = 1.68 Å^−1^ that appears for both nanocomposites with 2 and 3 wt % Na_0.33_WO_3_ TBNRs corresponds to the (001) peak of Na_0.33_WO_3_ TBNRs. The other three peaks at q = 1.52, 2.11, and 2.53 Å^−1^ (d = 4.13, 2.98 and 2.48 Å) only appear for the nanocomposite with 2 wt % Na_0.33_WO_3_ TBNR and originate from the formation of aggregates, as shown in Figure 7b. Thus, it becomes clear that the early breakdown of the nanocomposite films with 1 and 2 wt % TBNRs at less than 100% tensile elongation originates from the inhomogeneous distribution of TBNRs in the EPDM matrix and the resulting fracture development with stress concentration near the TBNR aggregates. In contrast, the 3 wt % Na_0.33_WO_3_ TBNRs that are relatively homogeneously distributed in the EPDM matrix as aggregates seemingly undergo rearrangement along the uniaxial direction, thereby relieving stress in the matrix and becoming elongated by more than 100%.

This rearrangement of the Na_0.33_WO_3_ TBNRs was further characterized by measuring 2D SAXS profiles with and without uniaxial elongation. To examine any changes in the SAXS profiles with the alignment of Na_0.33_WO_3_ TBNRs, we measured the 2D SAXS profiles of a pristine EPDM film and a nanocomposite film with 3 wt % Na_0.33_WO_3_ TBNRs without tensile elongation (Figure 7e,f, respectively). Then we measured SAXS profiles at tensile strains of 50%, 100%, and 200% (the maximum that we could reach), and integrated azimuthal cuts in the vertical (170–190° and −10° to 10°) and horizontal (80–100° and 260–280°) directions (Figure 7g). The horizontal direction is parallel with the tensile elongation direction. The 1D SAXS profiles after integration are shown in Figure 7h–j. The 2D and 1D SAXS profile of the pristine EPDM film show no distinct peaks and no change with and without tensile elongation, as shown in Figure 6e,h, even at 200% strain. In comparison, the 1D SAXS profile of the nanocomposite with 3 wt% Na_0.33_WO_3_ TBNR without uniaxial elongation contains a peak at the same position at around *q* = 0.164 Å^−1^ in both the horizontal (*d* = 38.42 Å) and vertical directions (*d* = 38.36 Å). However, at 200% strain, the maximum strain for a nanocomposite film with 3 wt% Na_0.33_WO_3_ TBNR, the peak in the horizontal direction was shifted to a low-*q* region of *q* = 0.16339 Å^−1^ (*d* = 38.56 Å) and the peak position in the vertical direction was *q* = 0.16467 Å^−1^ (*d* = 38.17 Å), a displacement of about 0.2 Å (Figure 5k). Because the fully extended length of OA is about 2 nm, a length of 3.8 nm is comparable to a distance between Na_0.33_WO_3_ TBNRs that are closely associated and where OA molecules are slightly interlocked. Thus, this increase in the distance between Na_0.33_WO_3_ TBNRs is proof of the separation between Na_0.33_WO_3_ TBNRs on tensile elongation. It should be mentioned that this peak shift also appears in the nanocomposites with 1 and 2 wt % Na_0.33_WO_3_ TBNRs (Figure S4), meaning that this separation between Na_0.33_WO_3_ TBNRs originates from their aggregates in the nanocomposites. We could not obtain any other distinct asymmetric 2D SAXS profiles in the *q*-range corresponding to the scale of the Na_0.33_WO_3_ TBNR lengths and diameters upon tensile elongation by the alignment of TBNRs that are independently dispersed in the matrix, as demonstrated by the anisotropic two-dimensional X-ray diffraction patterns of a polyurethane matrix with 10 wt % ZnO nanorods [46] possibly because the Na_0.33_WO_3_ TBNR contents in our nanocomposites are low and the aspect ratio of particular aggregates is not large.

The aggregation of Na_0.33_WO_3_ TBNRs with OA as grafted chains in a chemically identical polymer matrix, EPDM, is the well-known autophobic dewetting phenomenon [56,57]. When the matrix degrees of polymerization (P) are larger than those of the grafts on nanorod surfaces (N) (i.e., α = P/N > 2 for nanorods, the case in our study) [58,59], the grafts chains experience entropic stretching penalties in the matrix. Thus, nanorods can aggregate with each other to reduce the stretching of the grafts, resulting in graft-graft interactions, excluding the nanorod aggregates from the matrix polymer chains with high molecular weights. Our results show that the autophobic dewetting is severe in the EPDM nanocomposites with 1 and 2 wt % Na_0.33_WO_3_ TBNRs and that the entropy penalties can be overcome with evenly distributed particular aggregates of 3 wt % TBNRs because mixing entropy favors dispersion of Na_0.33_WO_3_ TBNRs at the increased filler concentration. The degree of aggregation with increasing Na_0.33_WO_3_ TBNR concentrations should be further thermodynamically elucidated as an independent research. In addition, site-to-site contacts between nanorods can be broken under tensile elongation, relieving the energy concentrated on nanorods aggregates, thereby enhancing the mechanical strain, as recently shown in a molecular dynamic simulation for nanorod aggregates [60].

## 4. Conclusions

In this work, we synthesized nanorods and nanoparticles of tungsten bronzes coated with alkyl (oleylamine) chains and prepared EPDM nanocomposites by taking advantage of the chemical structure of the olelylamine coating, which is similar to that of the EPDM matrix. We have demonstrated that the resulting nanocomposite films show a significant photothermal response (temperature increase) under white light and NIR light irradiation owing to the NIR absorption by the tungsten bronze nanomaterials, as well as improved mechanical behavior, which was modulated by the morphologies of the TBNs, compared to the pristine EPDM. Notably, the nanocomposite film of EPDM with 3 wt % Na_0.33_WO_3_ TBNRs presented an efficient photothermal temperature increase at a rate of 3.5 °C/min, reaching 40 °C in 5 min under NIR irradiation by a commercial NIR lamp, suggesting its application to photothermal skin patches. It also showed an enhanced tensile strain at break (165%) in comparison to that of the pristine EPDM (120%) because of the rearrangement of the nanorods under uniaxial elongation. These noticeable enhancements in both the photothermal and tensile strain behaviors make the EPDM nanocomposite films with TBNRs promising materials for applications that require cold weather resistance and photothermal heat generation for enhanced transdermal delivery and liquid droplet movement. Moreover, they are economic, yielding property enhancements at only a few weight percent filler loading. Further systematic, thermodynamic studies for rubber nanocomposites including our functional nanorods would result in a new class of elastomers with enhanced dynamic and mechanical performance together with the photothermal property.

## Figures and Tables

**Table 1:** Phase transition data and mechanical properties of pristine EPDM and nanocomposite films.

Sample	DMA		DSC	Tensile		Photothermal Temp.	
	E′ (at 20 °C) (MPa)	*T*_g_ (tan*δ*) (°C)	*T*_g_ (°C)	Strength (MPa)	Strain (%)	Max. ^a^ (°C)	Rate ^b^ (°C/min)
Pristine EPDM	1.76	−40.4	−48.3	0.59 ± 0.03	120 ± 16	22.3	0.4
1 wt% TBNR	2.14	−40.8	−47.2	0.55 ± 0.11	51 ± 6	31.9	2.0
2 wt% TBNR	3.97	−41.0	−48.2	0.40 ± 0.06	61 ± 18	39.2	3.3
3 wt% TBNR	2.52	−38.8	−47.3	0.61 ± 0.05	165 ± 20	41.0	3.5
1 wt% TBNP	–	–	−46.9	0.63 ± 0.10	130 ± 21	34.2	2.9
2 wt% TBNP	–	–	−47.8	0.60 ± 0.08	93 ± 14	39.8	3.3
3 wt% TBNP	–	–	−46.5	0.89 ± 0.16	98 ± 20	42.1	3.7

^a^ Maximum temperature increase after irradiation with white light for 1 h, ^b^ Rate of photothermal temperature increase in the first 3 min after irradiation with white light.

## Supplementary Materials

The following are available online at https://www.mdpi.com/2073-4360/11/11/1740/s1, Figure S1: X-ray diffraction patterns of (a) Na_0.33_WO_3_ and (b) Cs_0.33_WO_3_. (c) TEM image of Cs_0.33_WO_3_ TBNPs, Figure S2: EDS spectrum of Na_0.33_WO_3_ nanorods, Figure S3: TEM image of rubber nanocomposites with 3 wt% Na_0.33_WO_3_ TBNRs, Figure S4: 1D SAXS profiles obtained by integrating azimuthal cuts of 2D images (vertical: 80–100° and 260–280°, horizontal: 170–190° and −10 to 10°): EPDM nanocomposites with (a) 1 wt% and (b) 2 wt% Na_0.33_WO_3_ TBNR at 100% elongation, Table S1: Compositions of tungsten bronze nanoparticles estimated by energy dispersive spectrometer.
