# Supplementary material for: Photothermal Polymer Nanocomposites of Tungsten Bronze Nanorods with Enhanced Tensile Elongation at Low Filler Contents
Source: Polymers (Basel). 2019 Oct 24;11(11):1740. doi: 10.3390/polym11111740 (PMC6918126; doi:10.3390/polym11111740)
Supplement: Supplementary file 1 [file polymers-11-01740-s001.pdf]

Supporting Information

Photothermal Polymer Nanocomposites of Tungsten Bronze Nanorods with Enhanced Tensile Elongation at Low Filler Contents

Byoungyun Jeon,[1] Taehyung Kim,[1] Dabin Lee,[1] Tae Joo Shin,[2] Kyung Wha Oh,[3,*] and Juhyun Park[1,*]

[1] School of Chemical Engineering and Materials Science, Institute of Energy Converting Soft Materials, Chung-Ang University, Seoul 06974, Republic of Korea; jpark@cau.ac.kr

[2] UNIST Central Research Facilities & School of Natural Science, Ulsan National Institute of Science and Technology (UNIST), Ulsan 44919, Republic of Korea

[3] Department of Fashion Design, College of Art, Chung-Ang University, Seoul 06974, Republic of Korea

* Correspondence: jpark@cau.ac.kr (J.P.); kwhaoh@cau.ac.kr (K.W.O.)

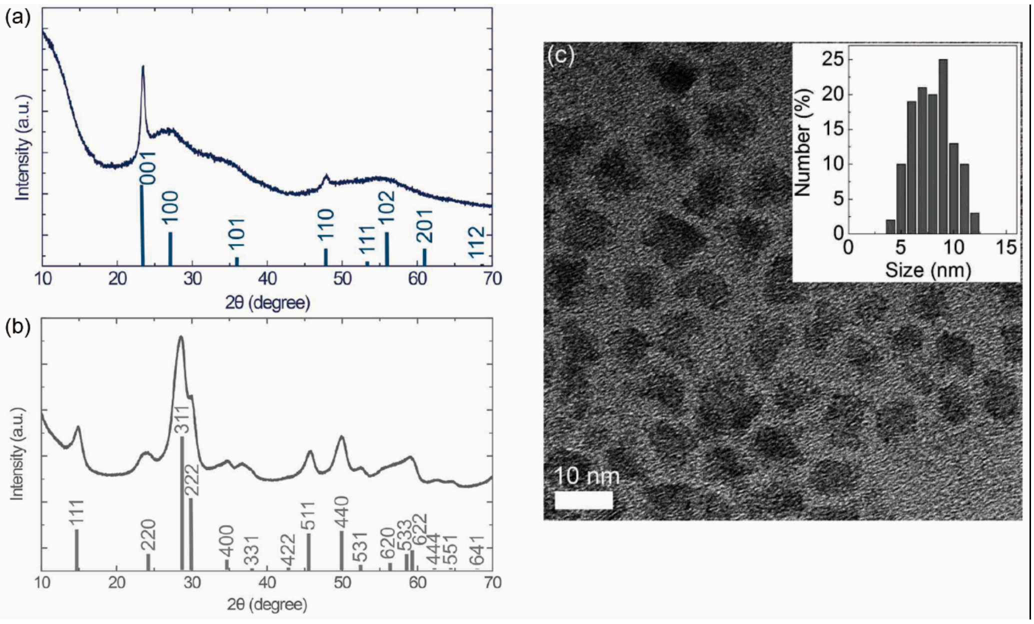

Figure S1. X-ray diffraction patterns of (a) $Na_{0.33}WO_3$ and (b) $Cs_{0.33}WO_3$. (c) TEM image of $Cs_{0.33}WO_3$ TBNPs.

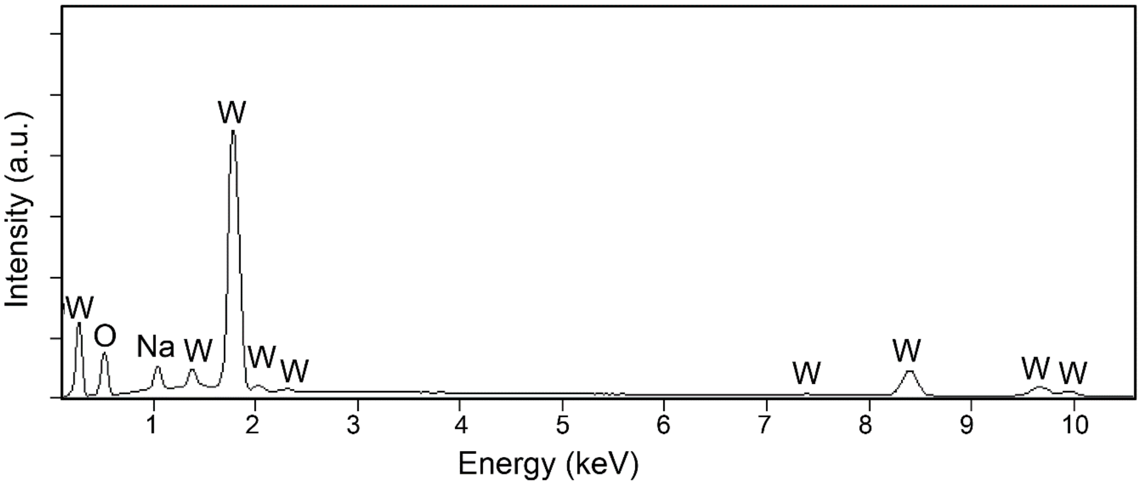

Figure S2. EDS spectrum of $Na_{0.33}WO_3$ nanorods.

Table S1. Compositions of tungsten bronze nanoparticles estimated by energy dispersive spectrometer

Sample	Elements	O-K	Na-K	W-L
$Na_{0.33}WO_3$	Mass (%)	20.64	3.25	76.11
	Mol (%)	3.11	0.34	1

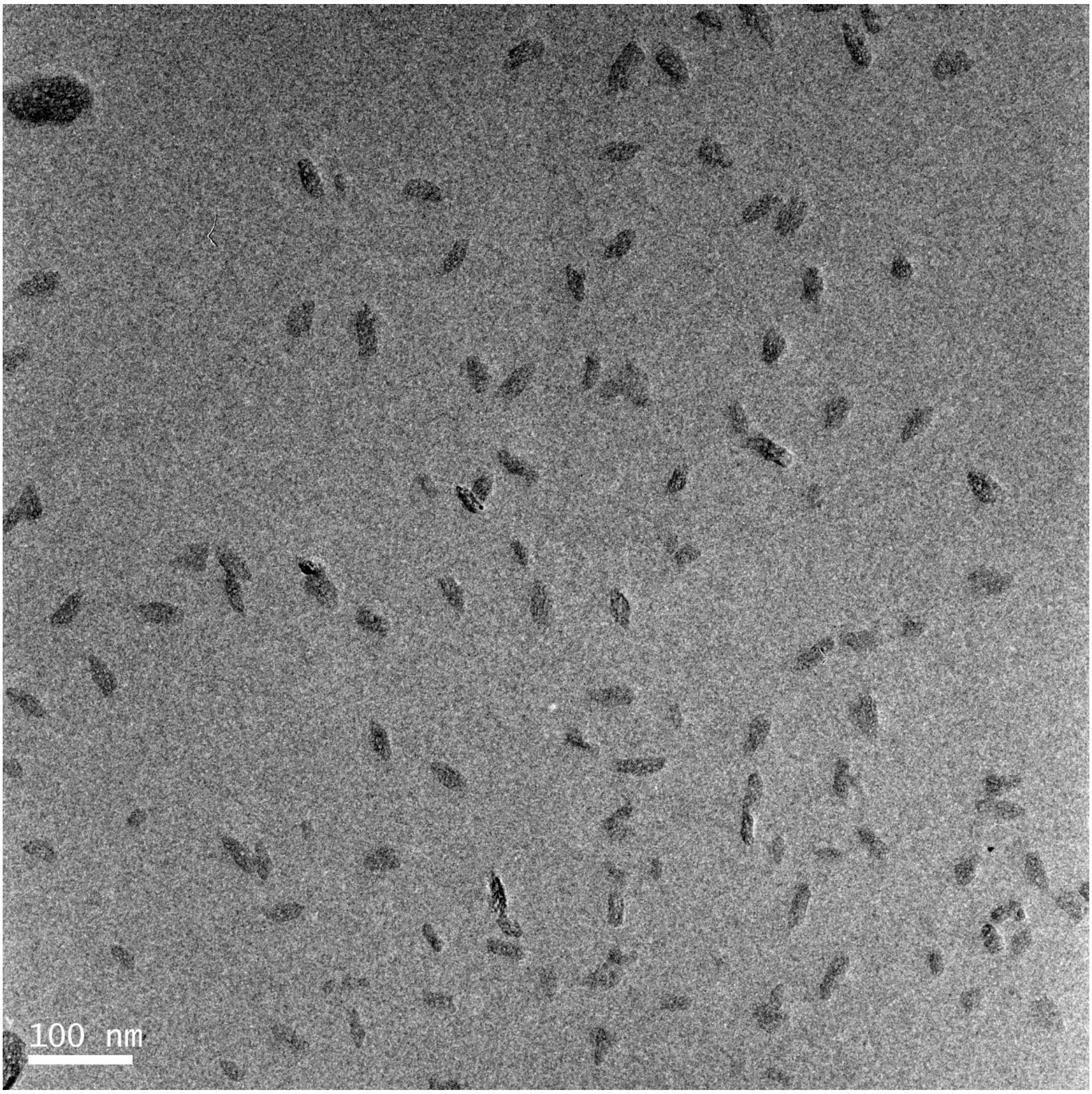

Figure S3. TEM image of rubber nanocomposites with 3-wt% $Na_{0.33}WO_3$ TBNRs.

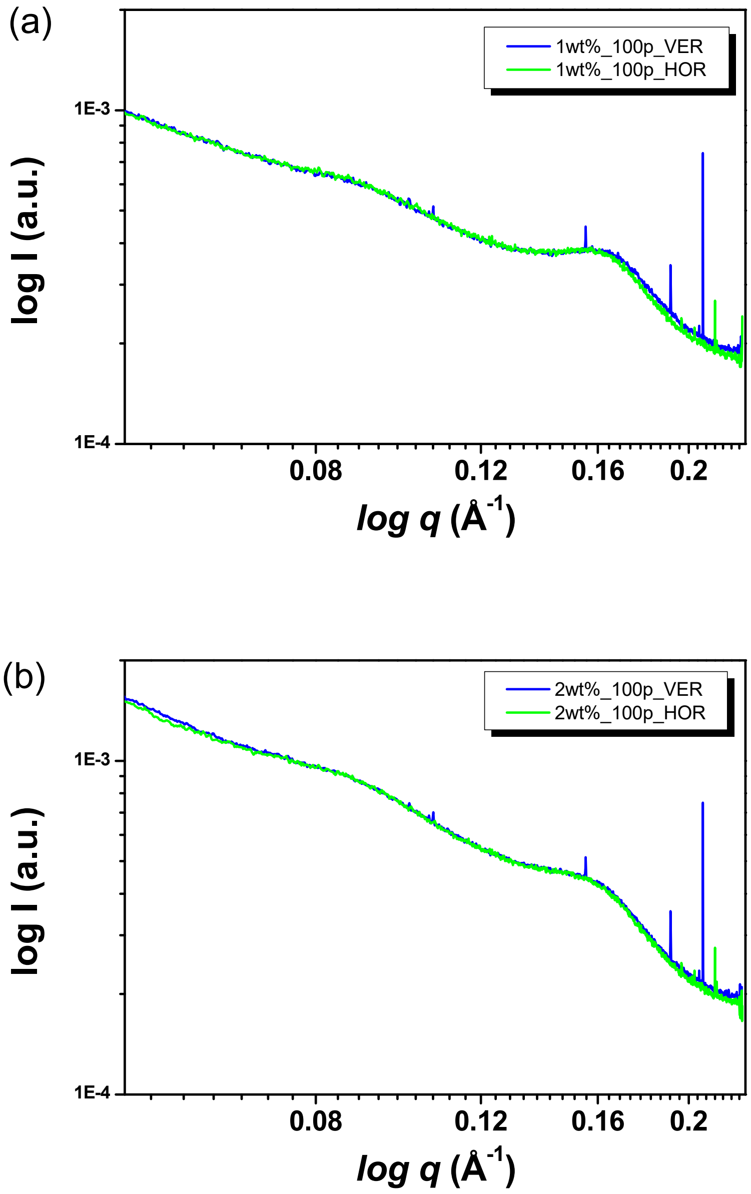

Figure S4. 1D SAXS profiles obtained by integrating azimuthal cuts of 2D images (vertical: 80–100º and 260–280°, horizontal: 170–190° and –10 to 10°): rubber nanocomposites with (a) 1-wt% and (b) 2-wt% $Na_{0.33}WO_3$ TBNR at 100% elongation.
